# Evaluation of Four Commonly Used DNA Barcoding Loci for *Ardisia* Species Identification

**DOI:** 10.3389/fpls.2022.860778

**Published:** 2022-04-07

**Authors:** Chao Xiong, Wei Sun, Lan Wu, Ran Xu, Yancheng Zhang, Wenjun Zhu, H. E. J., Panjwani, Zhiguo Liu, Bo Zhao

**Affiliations:** ^1^School of Life Science and Technology, Wuhan Polytechnic University, Wuhan, China; ^2^Institute of Chinese Materia Medica, China Academy of Chinese Medical Sciences, Beijing, China; ^3^Department of Pharmacognosy, Pharmacy School, Guilin Medical University, Guilin, China; ^4^Research Institute of Chemistry, International Center for Chemical and Biological Sciences, University of Karachi, Karachi, Pakistan; ^5^Center for Molecular Medicine and Drug Research, International Center for Chemical and Biological Sciences, University of Karachi, Karachi, Pakistan

**Keywords:** *Ardisia*, DNA barcoding, species identification, ITS fragment, cpDNA fragment

## Abstract

*Ardisia* plants have been used as medicinal plants for a long time in China. Traditional techniques such as morphological, microscopic, and chemical identification methods all have limitations in the species identification of *Ardisia*. For the sake of drug safety, four DNA barcodes (*psbA-trnH*, ITS, *rbcL*, and *matK*) were assessed for Chinese *Ardisia* plants using a total of 121 individuals from 33 species. Four criteria (The success rates of PCR amplification, DNA barcoding gap, DNA sequence similarity analysis and NJ tree clustering analysis) were used to evaluate the species identification ability of these four DNA barcodes. The results show that ITS had the highest efficiency in terms of PCR and sequencing and exhibited the most apparent inter- and intra-specific divergences and the highest species identification efficiency. There was no significant increase in species identification after combining the three cpDNA fragments with the ITS fragment. Considering the cost and experimental effectiveness, we recommend ITS as the core barcode for identifying Chinese *Ardisia* plants.

## Introduction

*Ardisia,* which comprises approximately 500 species worldwide and 65 species, have been recorded in the latest publication of “Flora of China” (Chen and Pipoly, 1996). *Ardisia* species have been used as medicine, food and ornamental plants for a long time. Because of their high medicinal and aesthetic value, *Ardisia* species have a sizeable exploratory potential and a broad market foreground (Liu et al., 2013). The dried plants of several *Ardisia* species have been used as Chinese Traditional Medicine for the treatment of ailments such as conjunctivitis, bronchitis, pneumonia, tuberculosis trauma, as well as pancreatic and other types of cancer (Zhao et al., 2014; François et al., 2016; Oliveira et al., 2018; Sanjeev et al., 2019). For example, the herb Aidicha found in China, the whole dried plant of *Ardisia japonica* (Thunberg) Blume, is officially listed in the Chinese Pharmacopoeia.

Since the 1970s, extensive research on the chemical components and pharmacological action of *Ardisia* has resulted in the discovery of many novel biologically active ingredients. Kobayashi and de Mejía (2005) have reviewed the chemical composition, biological activity, and pharmacological effects of many *Ardisia* plants. *Ardisia* species contain various physiologically active compounds, such as peptides, saponins, isocoumarins, quinones and alkylphenols I; therefore, it has high medicinal value with antitussive, anti-fertility, antiasthmatic, anti-inflammatory, antibacterial, anti-viral, anti-tumor and insecticidal effects (Newell et al., 2010; Joaquín-Cruz et al., 2015). Based on these pharmacological, now various products such as Aidicha Capsules, Compound Aidicha Tablets and Zouchuan Guci Ding have already been produced and used in clinical applications in China (Xin et al., 2015); in addition, extracts of *Ardisia colorata* Roxb are also commonly used in Thailand to treat gastrointestinal infections (Voravuthikunchai et al., 2004).

Despite a long history of its medicinal use in China, the taxonomic ambiguities make proper identification and acquisition of plant materials of some *Ardisia* species difficult. Mistaken identification is also due to either confusing nomenclature or several common terms with transliterated and local names, to the extent that some areas have 5 or 6 different names for the same species. Besides taxonomic confusion, the safety and quality of *Ardisia* herbal products have been a matter of increasing concern (Liu et al., 2013). Manufacturers may be tempted to label their food products incorrectly and add lower-priced ingredients of inferior quality to increase their profit because suspect or counterfeit herbal materials have been found on sale. Market surveys identified the low-cost herbs *Rhododendron molle* root and *Clerodendrum cyrtophyllum* stem as the primary adulterated materials in the commercial products of *A. gigantifolia* (Dai et al., 2018). Therefore, the safety and quality of *Ardisia* herbal products need to be urgently addressed to protect customer health and maintain the quality and authenticity of these herbal products in the drug supply chain.

While morphological, microscopic, and chemical identification methods are primarily used to authenticate herbal materials from *Ardisia*, all of these traditional techniques have limitations. Besides the morphological similarity and variation in sample profiles, the accuracy of these methods also lies in the assessor’s expertise. In addition, convergent evolution and extensive intra-species morphological variation make it laborious to identify and classify *Ardisia* species. It is also challenging to identify the botanical origin when analyzing heavily processed plant material. Recent developments in molecular biology and molecular genetic techniques have enabled the identification and authentication of *Ardisia* species. DNA-based methods are widely used in different research fields because they are rapid and sensitive. DNA barcoding, developed by the Centre for Biodiversity Genomics at the University of Guelph (Canada), is a powerful tool for species identification (Hebert et al., 2003a,b). This method is not limited by physiological conditions and morphological characteristics of samples, allowing species identification even without specialized taxonomic knowledge. The method can also be standardized for specific DNA barcodes and universal primers, a characteristic that is advantageous for building databases and creating a universal standard for identification (Chen et al., 2010; Yao et al., 2010; Li et al., 2011; Poudel et al., 2011). This method can identify species rapidly and accurately from a broad range and variable quality of raw materials and has a huge application potential in the food and medicine industries, which mainly ensures the use of correct, uncontaminated, and unsubstituted herbal ingredients (Chen et al., 2014). DNA barcode has become one of the most important tools for medicinal plant taxonomy and is used to identify adulterants in commercial herbal products (Cui et al., 2020; Yang et al., 2020), could regulate the quality in raw herbal trade market (Santhosh Kumar et al., 2015; Skjua et al., 2020).

Variation within a standard region of the genome called “DNA barcode” is analyzed using the DNA barcoding approach. This short sequence, which is derived from a suitable segment of the mitochondrial, chloroplast, or nuclear genome, is used to identify organisms at the species level. In 2009, after analyzing seven plastid DNA regions, including *atp*F*-atp*H*, mat*K, *rbc*L, *rpo*B, *rpo*C1, *psb*A*-trn*H, and *psb*K*-psb*I in 907 samples of 550 species, a combination of chloroplast Maturase K (*mat*K) and ribulose-bisphosphate carboxylase (*rbc*L) has been recommended as the core barcode for land plants (CBOL Plant Working Group, 2009). Subsequently, the chloroplast *psb*A-*trn*H region and the internal transcribed spacer (ITS) of nuclear ribosomal DNA were also considered for the core barcode of seed plants (Fazekas et al., 2008; China Plant BOL Group, 2011; Amritha et al., 2020; Zhang and Jiang, 2020). Usually, in plants, while the *mat*K region and the intergenic spacer *psb*A-*trn*H have evolved rapidly, the evolution of the *rbc*L region has not been so swift. In addition, a barcoding locus, the ITS region was more conservative than them, and all of them have been effectively used for complex plant groups. Although single or combined loci were used for candidate barcode sequences for plant identification, the most suitable DNA barcode for specific groups must be chosen by sequencing and analysis (Hollingsworth et al., 2011).

In *Ardisia*, the development of DNA barcoding is still nascent with few studies examining DNA regions to discriminate between species. This study used four core DNA barcodes (ITS, *mat*K, *rbc*L and *psb*A*-trn*H) to identify Chinese *Ardisia* plants. In this study, we aimed to assess the utility of these regions in Chinese *Ardisia* species and identify and screen out the best sequence suitable for applying DNA barcode technology in Chinese *Ardisia* species discrimination.

## Materials and Methods

### Taxon Sampling

Based on the field investigation, 121 samples from 33 wild Chinese *Ardisia* species were included in this study. For molecular analyses, fresh leaves were randomly collected in the squaring period and desiccated in silica gel. *Embelia laeta*, *E. rudis* and *Glaux maritima* were selected as outgroups. The *Ardisia* species used in this study are listed in Supplementary Table 1. We verified the identity of all of the samples independently through consultation with expert Shizhong Mao, who is an Associate Researcher at the Guangxi Institute of Botany, Chinese Academy of Sciences. Voucher specimens have been deposited in the Guangxi Institute of Botany, Chinese Academy of Sciences.

### DNA Isolation, Amplification and Sequencing

For each sample, 30–40 mg of leaves dried by silica gel were used, and genomic DNA was extracted and purified according to the Plant Genomic DNA Kit (Tiangen Biotech Co., China). The DNA concentration was estimated using BioTek Epoch (BioTek, Co., United States) by standard spectrophotometric methods at 260 and 280 nm. DNA integrity was assessed by electrophoresis using 1.0% agarose gel. Then, the DNA samples were diluted to a working concentration of 50 ng/μl and stored at −20°C until further use. According to Zhang et al. (2012), four commonly used DNA barcoding loci (*mat*K, *rbc*L, *psb*A*-trn*H, and ITS) were used in this study. The steps were carried out according to the China Plant BOL Group (2011) and Chen et al. (2010) using DNA barcoding standard operating procedures (DNA barcoding SOP).

PCR amplification was performed in 25 μl reaction mixtures containing 20–50 ng of genomic DNA, 12.5 μl of 2 × Taq PCR MasterMix (Beijing Aidlab Biotech Co., Beijing, China), 1 μl of 2.5 μM forward and reverse primers, and distilled water up to the final volume. PCR products were assessed on 1.0% agarose gel, visualized under UV light, purified using a Multifunction DNA Purification Kit from Bioteke (China) and then sequenced in both directions on a 3730XL sequencer (Applied Biosystems, United States) using amplification primers listed in Supplementary Table 2 (Chen, 2015).

### Sequence Analyses

The sequences were proofread, assembled as contigs and consensus sequences were generated using the CodonCode Aligner 4.2.1 (CodonCode Co., Dedham, MA, United States). Then, the Basic Local Alignment Search Tool (BLAST, NCBI) was used to check the homology of the obtained sequences. The CLUSTAL X 2.0 (Larkin et al., 2007) was applied using the default parameters and then manually rectified for multiple nucleotide alignment. The base compositions, the genetic distances, variable sites and parsimony-informative site values were estimated by MEGA5.1 as per the K2P (Kimura 2 parameter) model (Tamura et al., 2011). Barcoding gaps were estimated by comparing the distributions of intra- and inter-specific divergences of each candidate locus using the program MEGA5.1.

The degree of species resolution (identification) for the four DNA barcode regions was evaluated using the NJ tree method. For each sequence data set, pairwise genetic distances and all possible combinations for the five sequence data sets were determined by the K2P (Kimura 2-parameter) method (Kimura, 1980) using MEGA5.1. Support for clades was evaluated by bootstrap analysis with 1,000 replicates. Species discrimination was considered successful only when a single clade in NJ (Neighbour Joining) trees with a bootstrap value above 50% was specifically formed by all conspecific individuals (Zhang et al., 2012).

Based on the analysis of DNA sequence similarity results, the Taxon DNA method was used to assess each barcode region and their probable combinations to determine the degree of species resolution they presented (Meier et al., 2006). Additionally, the “best match” and the “best close match” functions and the Taxon DNA method were applied to test the individual-level discrimination rates for each single marker and all possible combinations under the K2P-corrected distance model. The “best match” was used to search the closest barcode match for each query. The identification was deemed successful if both sequences were from the same species, whereas mismatched names were failures. However, if there were several equally valid ‘best matches’ from different species, they were considered ambiguous. The “best close match” was used to plot the relative frequency of intraspecific distances, and the threshold value less than 95% of all intraspecific distances was set. Each query that did not have a barcode match below the threshold value could not be identified. For the remaining queries, their identities were compared with the species identities of their closest barcodes. If the name was identical, the query was considered successful identification. The query was considered a failure when the names were mismatched and ambiguous when several equally valid best matches belonged to a minimum of two species (Meier et al., 2006; Zhang et al., 2012).

Finally, the NCBI BLAST program 2.2.29 + (Tao, 2010) was used for all sequences analyzed using the “BLASTn” command to build local reference databases. Successful species discrimination was deemed when all species had the highest hit matching only a conspecific individual; for better clarity, the query sequence was removed from the list of top hits (Meyer and Paulay, 2005).

## Results

### PCR and Sequence Analysis

Total genomic DNA was extracted from 121 samples representing 33 Chinese Ardisia species, and then PCR and sequencing were carried out. All 468 sequences, including those of 119 matk, 114 rbcL, 121 ITS, and 114 trnH-psbA sequences, were obtained in this study, and submitted to the GenBank database (Supplementary Table 1). The efficiency of PCR amplification, in descending order, was 100.00% (ITS), 98.35% (matK), 94.21% (rbcL), and 94.21% (psbA-trnH). The failed species for matK were *A. arborescens*. The failed species for rbcL and psbA-trnH were *A. arborescens*, *A. humilis,* and *A. obtusa*. The sequencing for all four loci had a 100.0% success rate (Table 1).

**Table 1:** Success rates for PCR amplification and sequencing, and sequence characteristics of each single candidate barcodes.

	ITS	*mat*K	*rbc*L	*psb*A-*trn*H
Number of samples (individuals)	121	119	114	114
Success rates for PCR amplification (%)	100	98.35	94.21	94.21
Success rates for sequencing (%)	100	100	100	100
Length range (bp)	599–611	848–856	705	378–454
Aligned sequence length (bp)	619	856	705	533
GC content (%)	54.9–58.8	32.4–34.5	43.0–43.7	25.6–28.0
No. variable sites	229	87	40	85
No. parsimony information variable sites	158	39	19	47
Mean inter-specific distance (range), %	0.09 (0–1.93)	0.03 (0–0.59)	0.08 (0–0.85)	0.11 (0–6.56)
Mean intra-specific distance (range), %	3.43 (0–7.59)	0.39 (0–1.59)	0.37 (0–1.41)	1.49 (0–7.56)

The summary of the sequence characteristics of the four regions is presented in Table 1. The ITS sequences were 599–611 bp long, with 54.9–58.8% GC content and its multiple sequence alignment consisted of 619 characters, while 158 of 229 variable sites were potentially informative of parsimony. The matK sequences were 848–856 bp long, with 32.4–34.5% GC content and its multiple sequence alignment consisted of 856 characters, while 39 of 87 variable sites were potentially informative of parsimony. The rbcL sequence was 705 bp long with 30.9% GC content and its multiple sequence alignment consisted of 705 characters, while 19 of 40 variable sites were potentially informative of parsimony. The psbA-trnH sequence was 378–454 bp long with 25.6–28.0% GC content and its multiple sequence alignment consisted of 533 characters, while 47 of 85 variable sites were potentially informative parsimony.

### Barcoding Gap Test

**Figure 1:**
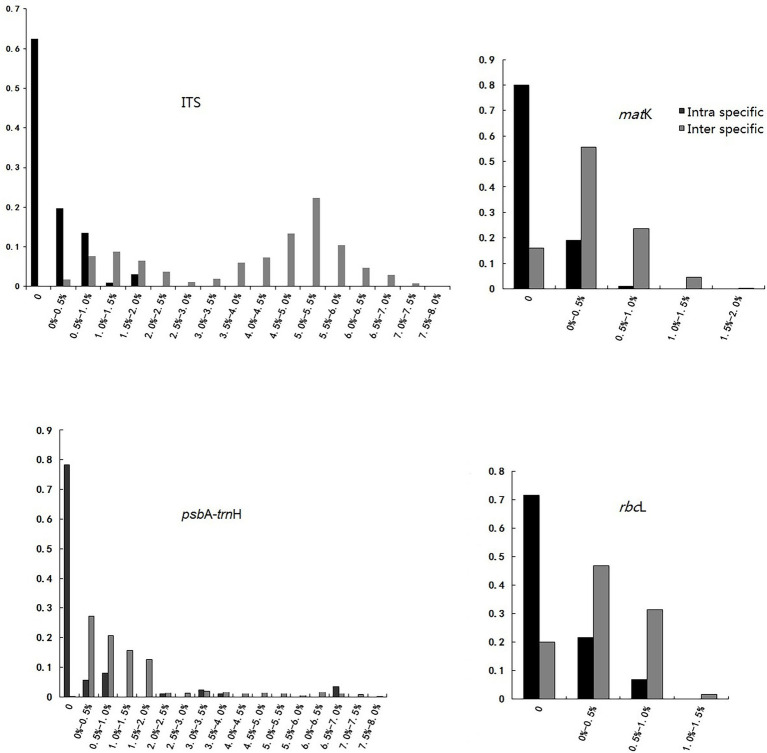
“Barcoding Gap” of each single candidate barcode.

In an ideal DNA barcode, the bar chart for divergence must show an intra-specific variation with the focus on the left side, having smaller numerals, while inter-specific variation should have the focus on the right side with greater numerals (Priyanka et al., 2015). This study observed obvious gaps between the intra- and inter-specific variability of ITS, although there was a slight overlap. For the other three loci, no evident barcoding gaps were found, and the overlap in the distributions of intra- and inter-specific variation was obvious (Table 1 and Figure 1).

### Species Discrimination Using NJ Tree Method and Local BLAST Method

The NJ tree method was used to assess the identification efficiency at the species level for Chinese Ardisia based on the four candidate barcodes (individually and in combination). If individuals of a species all formed a monophyletic clade, it was considered a successful identification at the species level. The results showed that species discrimination levels for the ITS markers and seven combinations containing ITS sequence were 79.3–83.8%, while species discrimination levels for the other three markers (matK, rbcL, and psbA-trnH) and four combinations without ITS sequence were less than 60%.

**Figure 2:**
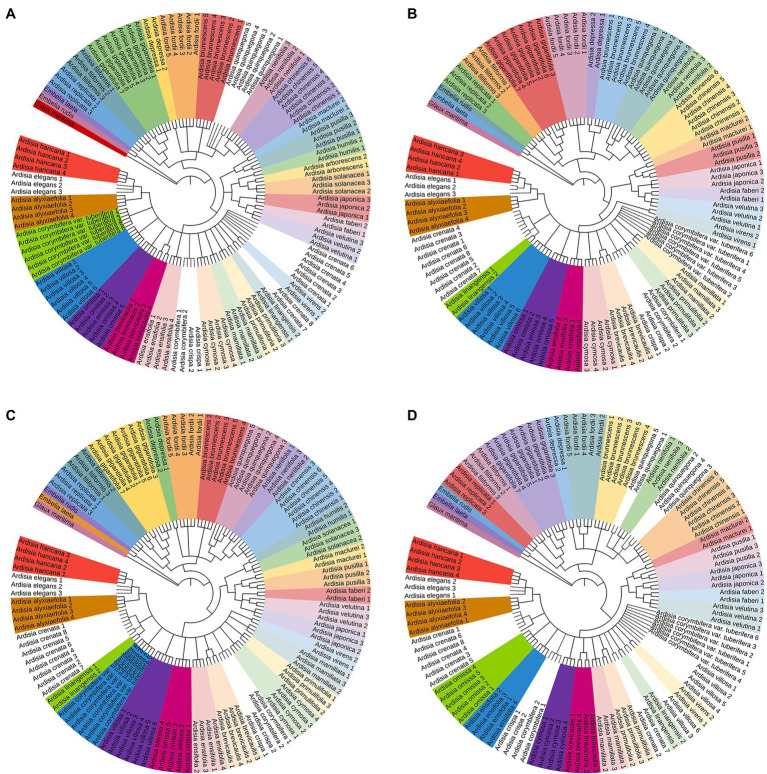
The NJ tree based on four sequences. Successfully identified species are with bootstrap values above 60%. The dotted line indicates unsuccessful identified species. **(A)** The NJ tree based on ITS sequences, **(B)** the NJ tree based on ITS + *psb*A-*trn*H sequences, **(C)** the NJ tree based on ITS + *mat*K sequences, **(D)** the NJ tree based on ITS + *rbc*L sequences.

**Figure 3:**
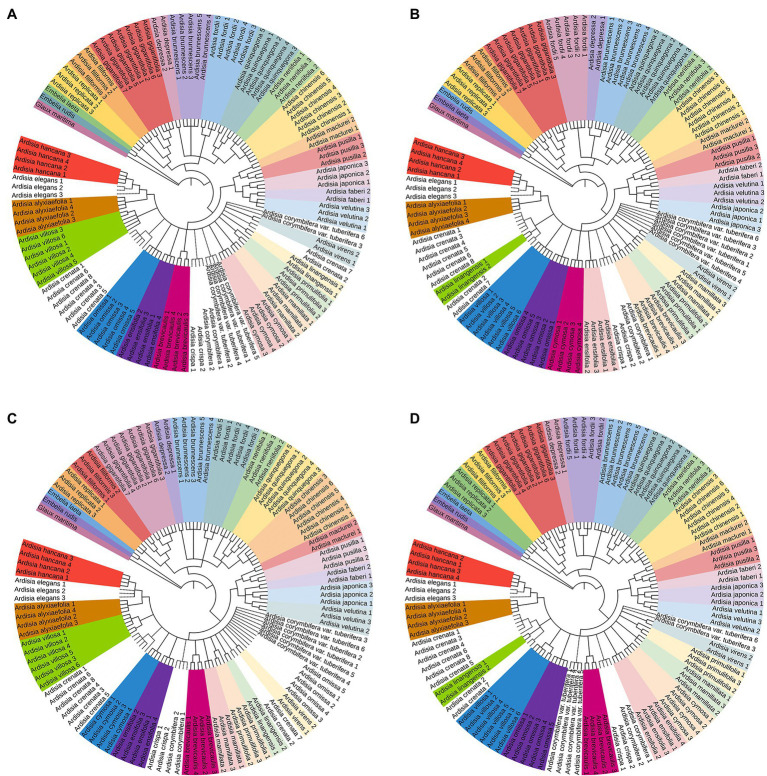
The NJ tree based on other four sequences. Successfully identified species are with bootstrap values above 60%. The dotted line indicates unsuccessful identified species. **(A)** The NJ tree based on ITS + *psb*A-*trn*H + *rbc*L sequences, **(B)** the NJ tree based on ITS + *mat*K + *psb*A-*trn*H sequences, **(C)** the NJ tree based on ITS + *mat*K + *rbc*L sequences, **(D)** the NJ tree based on ITS + *psb*A-*trn*H + *mat*K *+ rbc*L sequences.

In the NJ trees based on ITS markers and the barcodes combination containing ITS sequence (individually and in combination), four species (including *A. crenata*, *A. crispa*, *A. corymbifera* and *A. elegans*) were completely indistinguishable, while the other species were identified to different degrees (Figures 2, 3). In NJ tree based on ITS marker, A. quinquegona could not be identified at the species level (Figure 2A). In NJ trees based on ITS + psbA-tmH, ITS + psbA-tmH + rbcL and ITS + matK + psbA-tmH + rbcL, A. corymbifera *var.* tuberifera could not be identified (Figures 2B, 3A,D). In the NJ tree based on ITS + matK, *A. omissa* could not be identified (Figure 2C). In the NJ tree based on ITS + rbcL, A. corymbifera var. Tuberifera and *A. omissa* could not be identified (Figure 2D). In the NJ tree based on ITS + matK + psbA-tmH, A. corymbifera var. tuberifera and *A. mamillata* could not be identified (Figure 3B). In the NJ tree based on ITS + matK + rbcL, A. corymbifera var. tuberifera and *A. omissa* could not be identified (Figure 3C). These results suggest that the different combinations of ITS and the other three chloroplast markers could only increase the species discrimination ability for a few species. ITS was the most suitable barcode for species identification in Chinese Ardisia, and chloroplast markers were supplementary barcodes. The results of the local BLAST method were consistent with those of the NJ method (Table 2).

**Table 2:** Identification success rates obtained using NJ tree and local blast analysis methods for each single candidate barcodes and combinations of them.

Single candidate barcodes and combinations of them	NJ tree	Similarity-based
	Method^*^	Method (BLAST)
ITS	84.85% (28/33)	84.85% (28/33)
*mat*K	31.25% (10/32)	31.25% (10/32)
*psb*A-t*m*H	33.33% (10/30)	33.33% (10/30)
*rbc*L	16.67% (5/30)	16.67% (5/30)
ITS + *mat*K	84.38% (27/32)	84.38% (27/32)
ITS + *rbc*L	80.00% (24/30)	80.00% (24/30)
ITS + *psb*A-*tm*H	83.33% (25/30)	83.33% (25/30)
*mat*K + *psb*A-*tm*H	46.67% (14/30)	46.67% (14/30)
*mat*K + *rbc*L	33.33% (10/30)	33.33% (10/30)
*psb*A-*tm*H + *rbc*L	33.33% (10/30)	33.33% (10/30)
ITS + *mat*K + *psb*A-*tm*H	80.00% (24/30)	80.00% (24/30)
ITS + *mat*K + *rbc*L	80.00% (24/30)	80.00% (24/30)
ITS + *psb*A-*tm*H + *rbc*L	83.33% (25/30)	83.33% (25/30)
*mat*K + *psb*A-*tm*H + *rbc*L	43.33% (13/30)	43.33% (13/30)
ITS + *mat*K + *psb*A-*tm*H + *rbc*L	83.33% (25/30)	83.33% (25/30)

* *Based on the proportion of monophyletic species with > 60% bootstrapping*.

### Taxon DNA Method to Analyze the Identification Result

The Taxon DNA software used two standards, namely “Best match” and “Best close match,” to evaluate the species identification rate of four single gene fragments and 11 combinations of multiple gene fragments (Supplementary Table 3). The ITS fragment yielded the highest rate of species identification, successfully identified 115 samples at the species level, with a success rate of 95.04%. The species identification rate of the three cpDNA fragments and their combination were low. The success rates of species identification of the ITS fragment and three cpDNA combined fragments could not be significantly improved. The results were consistent with the analyses based on the NJ tree and the local BLAST method. The identification rate of the ITS fragment in Chinese Ardisia species was the highest. Hence, ITS could be used as the standard barcode for the genus Ardisia. Finally, the cpDNA gene fragments were used to compensate when individual species could not be identified by ITS.

## Discussion

### The Efficiency of PCR and Sequencing

A fundamental function of DNA barcode is to determine appropriate barcode sequences. After comprehensive screenings of gene regions of the plant genome, one nuclear (ITS) gene and three chloroplast genes (*rbc*L, *mat*K, and *trn*H*-psb*A) regions have been considered the core barcode in most plants (China Plant BOL Group, 2011; Hollingsworth et al., 2011). The efficiency of PCR amplification and the success rate of sequencing are important indicators for evaluating DNA barcode. In this study, the efficiency of PCR amplification, from high to low, was 100.0% (ITS), 98.35% (*mat*K), 94.21% (*rbc*L), and 94.21% (*psb*A*-trn*H). Three cpDNA fragments could not be successfully amplified in *A. arborescens*, *A. omissa*, and *A*. *obtusa* samples. The result might be caused by poor DNA template quality or any nucleotide variation in the primer binding region of the species.

### Species Identification Efficiency Using Three Chloroplast Regions

Plants of *Ardisia* were used as medicinal plants in China for a long time, but their similar morphological characteristics made them very difficult to differentiate. Liu et al. (2013) analyzed four markers (*psb*A-*trn*H, ITS2, *rbc*L, and matK) in 54 samples representing 24 species of the genus *Ardisia* to choose suitable DNA markers for authenticating and differentiating *Ardisia* at species level. But the sample numbers were limited to research, and most species had no repeated sampling. A small sample size of species may lead to underestimating intraspecific variation or overestimating interspecific differences without analysis of sister groups, which resulted in high validity and accuracy of DNA barcode identification in DNA barcode analysis results (Zhang et al., 2012; Yan et al., 2015). In this study, to identify suitable DNA markers, the sample size was increased to 121 samples representing 33 *Ardisia* species from China, with most species having two or three replicate samples.

The *rbc*L fragment is easily amplified and sequenced, and was chosen to identify flowering plants by Kress et al. (2005). The amplification rate of the *rbc*L primer for Chinese *Ardisia* species was almost 94% in this study, but the species identification rates in the three analysis methods were less than 20%. This result was consistent with the previous results according to which *rbc*L showed the lowest discrimination rate in seed plants (Lahaye et al., 2008; Ning et al., 2008). The *rbc*L sequences were not suitable as the DNA barcode sequences in Chinese *Ardisia*.

Non-coding regions have their advantages in the study of barcodes. For example, *psb*A-*trn*H is one of the fastest evolving segments in chloroplast fragments and is convenient for primer design (Hollingsworth et al., 2011). Although the length variation of *psb*A-*trn*H sequences was large, the species identification rate of this fragment in the NJ tree and local Blast method was less than 30%. Thus, *psb*A-*trn*H sequences were not suitable as the DNA barcode sequences in Chinese *Ardisia*.

Previous studies suggested that the species discrimination rate would drop significantly when *mat*K sequences were used to distinguish between closely related species or an extensively sampled genus (Ren et al., 2010; Yan et al., 2011). When 54 samples of 24 species from the genus *Ardisia* were analyzed, the results indicated that the *mat*K region was a promising DNA barcode, with the highest species identification efficiency at 91.7% by the nearest distance method and 98.1% using the basic local alignment search tool method. When 121 samples of 33 Chinese *Ardisia* species were used in our study, the amplification and sequencing success rate of *mat*K sequences for Chinese *Ardisia* species was 98.35%. Still, species identification rates obtained through the three analysis methods were less than 50%. However, the performance of *mat*K was overestimated when insufficient samples were used. The discrimination ability of *mat*K for Chinese *Ardisia* was low, and it was also not suitable as a barcode for the identification of Chinese *Ardisia* species.

When the identification rate of a single fragment failed to satisfy the requisites of their purpose, DNA barcode combinations could improve the species identification efficiency (CBOL Plant Working Group, 2009; Li et al., 2015; Yan et al., 2015). In this study, the effects of using a combination of chloroplast fragments gave limited improvement, consistent with the results in Lysimachia (Myrsinaceae) and Codonopsis (Campanulaceae) (Zhang et al., 2012; Wang et al., 2017). The species identification rates using the three cpDNA fragment combinations were less than 50%. Therefore, they are not suitable DNA barcodes for Chinese *Ardisia* species identification.

### Species Identification Efficiency of ITS

Internal transcribed spacer exhibited high species resolution and was proposed as the core barcode for seed plants (Li et al., 2015). While the cpDNA sequences in 5–10% of angiosperms were not easily amplified, the ITS sequences could be amplified more easily. This study obtained the same result that the amplification success rate of ITS was high, reaching 100%. In flowering plants, ITS sequences perform efficiently as molecular markers, with 3–4 times higher variation sites than cpDNA fragments (Zhang et al., 2012). Our results were consistent with those of previous studies. Compared to *mat*K, *rbc*L, and *psb*A*-trn*H the number of variation sites of ITS was almost four, nine, and four times higher, respectively. Fu et al. (2011) demonstrated that the identification rate of Vitaceae species was 93.8%. Wilcoxon signed-rank tests reveal that ITS was stronger than other core barcodes regardless of interspecific or intraspecific variations. Our results also suggested that the ITS fragment’s identification rate using three analysis methods in Chinese *Ardisia* species was higher than 80%, but closer to 100%. ITS showed the highest number of variation sites and the most efficient amplification, sequencing, and identification among the core barcodes. After combining three cpDNA fragments with ITS, the corresponding rate of species identification did not increase significantly using three analysis methods. Therefore, considering cost and experimental effectiveness, we recommend only ITS to identify Chinese *Ardisia* plants.

### Classification of Chinese *Ardisia* Species

Except for *A. crispa* and *A. corymbifera*, the other species were completely resolved by single or combination markers. Because the sequences of the two species are identical, samples of *A*. *crispa* and *A. corymbifera* formed a monophyletic clade in the NJ trees (Figures 2, 3), and these two species were not resolved. Other DNA barcoding loci should further analyze the unresolved species.

Samples of *A. crenata* and *A*. *linangensis*, formed a monophyletic clade in NJ trees based on ITS + *matK*, ITS + *psb*A*-tm*H, ITS + *mat*K + *psb*A*-tm*H and ITS + *mat*K + *psb*A-*tm*H + *rbc*L combination sequences. Two *A. linangensis* samples always formed a monophyletic clade in the NJ tree analysis. The results were consistent with the findings of Wang and Xia (2012, 2013). Therefore, we also did not support the idea that *A. crenata* var. *bicolor* should be merged with A. crenata to form the *A. crenata* complex. However, the samples were limited, and more samples should be collected and analyzed to determine the *A. crenata* complex.

## Conclusion

*Ardisia* plants have been mostly used for medicinal purposes in China for a long time, with good effects on rheumatism, phthisis and various kinds of inflammation. Due to the high degree of similarity in morphology and the phenomenon of homonymy, the traditional identification methods can not accurately identify the species. For the sake of drug safety, the species identification ability of four DNA barcodes (*psb*A-*trn*H, ITS, *rbc*L, *mat*K) in *Ardisia* plants in China was evaluated in this study. The results showed that ITS showed the highest number of variation sites and the most efficient amplification, sequencing and identification. Using three cpDNA fragments alone, combined with each other or combined with ITS fragments, the efficiency of species identification did not significantly increase. Considering the cost and experimental effect, we recommend ITS as the core barcode for plant identification of *Ardisia* in China.

## Data Availability Statement

The datasets presented in this study can be found in online repositories. The names of the repository/repositories and accession number can be found in the article/Supplementary Material.

## Author Contributions

ZL and BZ supervised the whole project. CX and WS performed the major research and wrote the manuscript in equal contribution. LW provided the technical support and language editing support. RX, YZ, and WZ provided their professional expertise. HJ and Panjwani provided data analysis. All authors contributed to the article and approved the submitted version.

## Funding

This work was supported by National Key R&D Program of China from the Ministry of Science and Technology of China (No. 2021YFE0100900), the National Science Foundation of China (No. 81903758), and the Fundamental Research Funds for the Central public welfare research institutes (ZZ13-YQ-106).

## Conflict of Interest

The authors declare that the research was conducted in the absence of any commercial or financial relationships that could be construed as a potential conflict of interest.

## Publisher’s Note

All claims expressed in this article are solely those of the authors and do not necessarily represent those of their affiliated organizations, or those of the publisher, the editors and the reviewers. Any product that may be evaluated in this article, or claim that may be made by its manufacturer, is not guaranteed or endorsed by the publisher.

## References

[ref1] Amritha N. Bhooma V. Parani M. (2020). Authentication of the market samples of Ashwagandha by DNA barcoding reveals that powders are significantly more adulterated than roots. J. Ethnopharmacol. 256:112725. doi: 10.1016/j.jep.2020.112725, PMID: 32126246

[ref2] CBOL Plant Working Group (2009). A DNA barcode for land plants. Proc. Natl. Acad. Sci. U. S. A. 106, 12794–12797. doi: 10.1073/pnas.0905845106, PMID: 19666622 PMC2722355

[ref3] Chen S. L. (2015). Standard DNA Barcodes of Chinese Materia Medica in Chinese Pharmacopoeia. Beijing: Medical Science and Technology Press.

[ref4] Chen S. L. Pang X. H. Song J. Y. Shi L. C. Yao H. Han J. P.. (2014). A renaissance in herbal medicine identification: from morphology to DNA. Biotechnol. Adv. 32, 1237–1244. doi: 10.1016/j.biotechadv.2014.07.004, PMID: 25087935

[ref5] Chen J. Pipoly J. J. (1996). “Myrsinaceae,” in Flora of China. eds. Wu Z. Raven P. H. (Beijing: Science Press), 1–38.

[ref6] Chen S. L. Yao H. Han J. P. Liu C. Song J. Y. Shi L. C.. (2010). Validation of the ITS2 region as a novel DNA barcode for identifying medicinal plant species. PLoS One 5:e8613. doi: 10.1371/journal.pone.0008613, PMID: 20062805 PMC2799520

[ref7] China Plant BOL Group Li D. Z. Gao L. M. Li H. T. Wang H. Ge X. J.. (2011). Comparative analysis of a large dataset indicates that internal transcribed spacer (ITS) should be incorporated into the core barcode for seed plants. Proc. Natl. Acad. Sci. U. S. A. 108, 19641–19646. doi: 10.1073/pnas.1104551108, PMID: 22100737 PMC3241788

[ref8] Cui X. Li W. Wei J. Qi Y. Zheng X. (2020). Assessing the identity of commercial herbs from a Cambodian market using DNA barcoding. Front. Pharmacol. 11:244. doi: 10.3389/fphar.2020.00244, PMID: 32265692 PMC7105672

[ref9] Dai W. Dong P. Tian S. Mei Q. (2018). A pharmacognostical study on *Ardisia gigantifolia* and its adulterants. Med. Plant. 9, 39–44. doi: 10.19600/j.cnki.issn2152-3924.2018.04.011

[ref10] Fazekas A. J. Burgess K. S. Kesanakurti P. R. Graham S. W. Newmaster S. G. Husband B. C.. (2008). Multiple multilocus DNA barcodes from the plastid genome discriminate plant species equally well. PLoS One 3:e2802. doi: 10.1371/journal.pone.0002802, PMID: 18665273 PMC2475660

[ref11] François C. Hul S. Deharo E. Bourdy G. (2016). Natural remedies used by Bunong people in Mondulkiri province (Northeast Cambodia) with special reference to the treatment of 11 most common ailments. J. Ethnopharmacol. 191, 41–70. doi: 10.1016/j.jep.2016.06.003, PMID: 27282662

[ref12] Fu Y. M. Jiang W. M. Fu C. X. (2011). Identification of species within *Tetrastigma* (Miq.) Planch. (Vitaceae) based on DNA barcoding techniques. J. Syst. Evol. 49, 237–245. doi: 10.1111/j.1759-6831.2011.00126.x

[ref13] Hebert P. D. N. Cywinska A. Ball S. L. de Waard J. R. (2003a). Biological identification through DNA barcodes. P. Roy. Soc. B-Biol. Sci. 270, 313–321. doi: 10.1098/rspb.2002.2218, PMID: 12614582 PMC1691236

[ref14] Hebert P. D. N. Ratnasingham S. de Waard J. R. (2003b). Barcoding animal life: cytochrome *c* oxidase subunit 1 divergences among closely related species. P. Roy. Soc. B-Biol. Sci. 270(Suppl. 1), S96–S99. doi: 10.1098/rsbl.2003.0025, PMID: 12952648 PMC1698023

[ref15] Hollingsworth P. M. Graham S. W. Little D. P. (2011). Choosing and using a plant DNA barcode. PLoS One 6:e19254. doi: 10.1371/journal.pone.0019254, PMID: 21637336 PMC3102656

[ref16] Joaquín-Cruz E. Dueñas M. García-Cruz L. Salinas-Moreno Y. Santos-Buelga C. García-Salinas C. (2015). Anthocyanin and phenolic characterization, chemical composition and antioxidant activity of chagalapoli (*Ardisia compressa* k.) fruit: a tropical source of natural pigments. Food Res. Int. 70, 151–157. doi: 10.1016/j.foodres.2015.01.033

[ref17] Kimura M. (1980). A simple method for estimating evolutionary rates of base substitution through comparative studies of nucleotide sequences. J. Mol. Evol. 16, 111–120. doi: 10.1007/BF01731581, PMID: 7463489

[ref18] Kobayashi H. de Mejía E. (2005). The genus Ardisia: a novel source of health-promoting compounds and phytopharmaceuticals. J. Ethnopharmacol. 96, 347–354. doi: 10.1016/j.jep.2004.09.037, PMID: 15619551

[ref19] Kress W. J. Wurdack K. J. Zimmer E. A. Weigt L. A. Janzen D. H. (2005). Use of DNA barcodes to identify flowering plants. Proc. Natl. Acad. Sci. U. S. A. 102, 8369–8374. doi: 10.1073/pnas.0503123102, PMID: 15928076 PMC1142120

[ref20] Lahaye R. Van der B. M. Bogarin D. Warner J. Pupulin F. Gigot G.. (2008). DNA barcoding the floras of biodiversity hotspots. Proc. Natl. Acad. Sci. U. S. A. 105, 2923–2928. doi: 10.1073/pnas.0709936105, PMID: 18258745 PMC2268561

[ref21] Larkin M. A. Blackshields G. Brown N. Chenna R. McGettigan P. A. McWilliam H.. (2007). Clustal W and Clustal X version 2.0. Bioinformatics 23, 2947–2948. doi: 10.1093/bioinformatics/btm404, PMID: 17846036

[ref601] Li D. Z. Gao L. M. Li H. T. Wang H. Ge X. J. Liu J. Q.. (2011). Comparative analysis of a large dataset indicates that internal transcribed spacer (ITS) should be incorporated into the core barcode for seed plants. Proc. Natl. Acad. Sci. U. S. A. 108, 19641–19646. doi: 10.1073/pnas.1104551108, PMID: 22100737 PMC3241788

[ref602] Li X. Yang Y. Henry R. J. Rossetto M. Wang Y. Chen S. (2015). Plant DNA barcoding: from gene to genome. Biol. Rev. 90, 157–166. doi: 10.1111/brv.12104, PMID: 24666563

[ref22] Liu Y. M. Wang K. Liu Z. Luo K. Chen S. L. Chen K. L. (2013). Identification of medical plants of 24 *Ardisia* species from China using the *mat* K genetic marker. Pharmacogn. Mag. 9, 331–337. doi: 10.4103/0973-1296.117829, PMID: 24124285 PMC3793338

[ref23] Meier R. Shiyang K. Vaidya G. Ng P. K. L. (2006). DNA barcoding and taxonomy in Diptera: a tale of high intraspecific variability and low identification success. Syst. Biol. 55, 715–728. doi: 10.1080/10635150600969864, PMID: 17060194

[ref24] Meyer C. P. Paulay G. (2005). DNA barcoding: error rates based on comprehensive sampling. PLoS Biol. 3:e422. doi: 10.1371/journal.pbio.0030422, PMID: 16336051 PMC1287506

[ref25] Newell A. M. B. Yousef G. G. Lila M. A. Ramírez-Mares M. V. de Mejia E. G. (2010). Comparative in vitro bioactivities of tea extracts from six species of *Ardisia* and their effect on growth inhibition of hepg2 cells. J. Ethnopharmacol. 130, 536–544. doi: 10.1016/j.jep.2010.05.051, PMID: 20561930

[ref26] Ning S. P. Yan H. F. Hao G. Ge X. J. (2008). Current advances of DNA barcoding study in plants. Biodivers. Sci. 16, 417–425. doi: 10.3724/SP.J.1003.2008.08215

[ref27] Oliveira N. A. Santos O. Y. I. Lima A. B. Elisabete A. D. M. M. Frota A. G. (2018). Pharmacological effects of the isomeric mixture of alpha and beta amyrin from protium heptaphyllum: a literature review. Fund. Clin. Pharmacol. 33, 4–12. doi: 10.1111/fcp.12402, PMID: 30003594

[ref28] Poudel R. C. Li D. Z. Forrest A. (2011). High universality of matK primers for barcoding gymnosperms. J. Syst. Evol. 49, 169–175. doi: 10.1111/j.1759-6831.2011.00128.x

[ref29] Priyanka M. Amit K. Akshitha N. Daya N. M. Ashutosh S. Rakesh T.. (2015). DNA barcoding: an efficient tool to overcome authentication challenges in the herbal market. Plant Biotechnol. J. 14, 8–21. doi: 10.1111/pbi.12419, PMID: 26079154 PMC11388846

[ref30] Ren B. Q. Xiang X. G. Chen Z. D. (2010). Species identification of *Alnus* (Betulaceae) using nrDNA and cpDNA genetic markers. Mol. Ecol. Resour. 10, 594–605. doi: 10.1111/j.1755-0998.2009.02815.x, PMID: 21565064

[ref31] Sanjeev S. Murthy M. K. Devi M. S. Khushboo M. Renthlei Z. Ibrahim K. S.. (2019). Isolation, characterization, and therapeutic activity of bergenin from marlberry (*Ardisia colorata* Roxb.) leaf on diabetic testicular complications in Wistar albino rats. Environ. Sci. Pollut. R. 26, 7082–7101. doi: 10.1007/s11356-019-04139-9, PMID: 30648235

[ref32] Santhosh Kumar J. U. Krishna V. Seethapathy G. S. Senthilkumar U. Ragupathy S. Ganeshaia K. N.. (2015). DNA barcoding to assess species adulteration in raw drug trade of “bala” (genus: sida l.) herbal products in South India. Biochem. Syst. Ecol. 61, 501–509. doi: 10.1016/j.bse.2015.07.024

[ref33] Skjua B. Mr C. Gss D. Vk A. Rus B. Gr E. (2020). DNA barcoding of momordica species and assessment of adulteration in momordica herbal products, an anti-diabetic drug. Plant Gene 22:100227. doi: 10.1016/j.plgene.2020.100227

[ref34] Tamura K. Peterson D. Peterson N. Stecher G. Nei M. Kumar S. (2011). MEGA5: molecular evolutionary genetics analysis using maximum likelihood, evolutionary distance, and maximum parsimony methods. Mol. Biol. Evol. 28, 2731–2739. doi: 10.1093/molbev/msr121, PMID: 21546353 PMC3203626

[ref35] Tao T. (2010). Standalone BLAST setup for windows PC. Available at: http://www.ncbi.nlm.nih.gov/books/NBK52637/ (Accessed August 31, 2020).

[ref36] Voravuthikunchai S. Lortheeranuwat A. Jeeju W. Sririrak T. Phongpaichit S. Supawita T. (2004). Effective medicinal plants against enterohaemorrhagic escherichia coli o157:h7. J. Ethnopharmacol. 94, 49–54. doi: 10.1016/j.jep.2004.03.036, PMID: 15261962

[ref37] Wang D. Y. Wang Q. Wang Y. L. Xiang X. G. Huang L. Q. Jin X. H. (2017). Evaluation of DNA barcodes in *Codonopsis* (Campanulaceae) and in some large angiosperm plant genera. PLoS One 12:e0170286. doi: 10.1371/journal.pone.0170286, PMID: 28182623 PMC5300163

[ref38] Wang J. Xia N. H. (2012). *Ardisia crenata* Complex (Primulaceae) Studies Using Morphological and Molecular Data. ed. Mworia J. K. (Botany, London: InTech), 163–172.

[ref39] Wang J. Xia N. H. (2013). Quantitative analysis of morphological characters of *Ardisia crenata* complex (Primulaceae). J. Trop. Subtrop. Bot. 6, 543–548.

[ref40] Xin X. Yu D. Zhu L. Gu Z. X. Yuan L. Huang S. (2015). Qualitative and quantitative method for compound Aidicha tablets. Cent. South Pharma. 13, 410–413.

[ref41] Yan H. F. Hao G. Hu C. M. Ge X. J. (2011). DNA barcoding in closely related species: a case study of *Primula* L. sect. *Proliferae* Pax (Primulaceae) in China. J. Syst. Evol. 49, 225–236. doi: 10.1111/j.1759-6831.2011.00115.x

[ref42] Yan L. J. Liu J. Möller M. Zhang L. Zhang X. M. Li D. Z.. (2015). DNA barcoding of *rhododendron* (Ericaceae), the largest chinese plant genus in biodiversity hotspots of the himalaya–hengduan mountains. Mol. Ecol. Resour. 15, 932–944. doi: 10.1111/1755-0998.12353, PMID: 25469426

[ref43] Yang C. Q. Lv Q. Zhang A. B. (2020). Sixteen years of DNA barcoding in China: what has been done? What can be done? Front. Ecol. Evol. 8:57. doi: 10.3389/fevo.2020.00057

[ref44] Yao H. Song J. Y. Liu C. Luo K. Han J. P. Li Y.. (2010). Use of ITS2 region as the universal DNA barcode for plants and animals. PLoS One 5:e13102. doi: 10.1371/journal.pone.0013102, PMID: 20957043 PMC2948509

[ref45] Zhang D. Jiang B. (2020). Species identification in complex groups of medicinal plants based on DNA barcoding: a case study on *Astragalus* spp. (Fabaceae) from Southwest China. Conserv. Genet. Resour. 12, 469–478. doi: 10.1007/s12686-019-01119-6

[ref46] Zhang C. Y. Wang F. Y. Yan H. F. Hao G. Hu C. M. Ge X. J. (2012). Testing DNA barcoding in closely related groups of *Lysimachia* L. (Myrsinaceae). Mol. Ecol. Resour. 12, 98–108. doi: 10.1111/j.1755-0998.2011.03076.x, PMID: 21967641

[ref47] Zhao C. Q. Zhou Y. Ping J. Xu L. M. (2014). Traditional Chinese medicine for treatment of liver diseases: progress, challenges and opportunities. J. Integr. Med. 12, 401–408. doi: 10.1016/S2095-4964(14)60039-X, PMID: 25292339 PMC7128864

