# Supplementary material for: Evaluation of Four Commonly Used DNA Barcoding Loci for Ardisia Species Identification
Source: Front Plant Sci. 2022 Apr 7;13:860778. doi: 10.3389/fpls.2022.860778 (PMC9021757; doi:10.3389/fpls.2022.860778)
Supplement: Supplementary file 1 [file Data_Sheet_1.ZIP › Revised Supplementary files/Supplementary Table 1.docx]

**Supplementary Table 1** Original information with voucher information and GenBank accession numbers of four markers for all the samples of Chinese *Ardisia* used in this study.

| Group | NO. | Species name | Voucher information | GenBank Accession NO. | | | |
| --- | --- | --- | --- | --- | --- | --- | --- |
|  |  |  |  | *rbc*L | *mat*K | *psb*A-*trn*H | ITS |
| Ⅰ | 1 | *Ardisia alyxiaefolia* 1 | MSZ-M110 | MF045660 | MF045542 | MF037911 | KY963698 |
|  | 2 | *Ardisia alyxiaefolia* 2 | MSZ-M130 | MF045672 | MF045554 | MF037923 | KY963710 |
|  | 3 | *Ardisia alyxiaefolia* 3 | MSZ-M79 | MF045729 | MF045611 | MF037980 | KY963767 |
|  | 4 | *Ardisia alyxiaefolia* 4 | MSZ-SNH22 | MF045734 | MF045616 | MF037985 | KY963772 |
| Ⅱ | 5 | *Ardisia arborescens* 1 | MSZ-XQM10 |  |  |  | KY963779 |
|  | 6 | *Ardisia arborescens* 2 | MSZ-XQM83 |  |  |  | KY963780 |
| Ⅲ | 7 | *Ardisia brevicaulis* 1 | MSZ-JGXX | MF045646 | MF045528 | MF037897 | KY963684 |
|  | 8 | *Ardisia brevicaulis* 2 | MSZ-M346 | MF045724 | MF045606 | MF037975 | KY963762 |
|  | 9 | *Ardisia brevicaulis* 3 | MSZ-M115 | MF045663 | MF045545 | MF037914 | KY963701 |
|  | 10 | *Ardisia brevicaulis* 4 | MSZ-M132 | MF045673 | MF045555 | MF037924 | KY963711 |
| Ⅳ | 11 | *Ardisia brunnescens* 1 | MSZ-M207 | MF045705 | MF045587 | MF037956 | KY963743 |
|  | 12 | *Ardisia brunnescens* 2 | MSZ-TM1 | MF045735 | MF045620 | MF037986 | KY963776 |
|  | 13 | *Ardisia brunnescens* 3 | MSZ-M204 | MF045704 | MF045586 | MF037955 | KY963742 |
|  | 14 | *Ardisia brunnescens* 4 | MSZ-M198 | MF045702 | MF045584 | MF037953 | KY963740 |
|  | 15 | *Ardisia brunnescens* 5 | MSZ-AM52 | MF045634 | MF045515 | MF037885 | KY963671 |
| Ⅴ | 16 | *Ardisia chinensis* 1 | MSZ-M117 | MF045664 | MF045546 | MF037915 | KY963702 |
|  | 17 | *Ardisia chinensis* 2 | MSZ-M332 | MF045715 | MF045597 | MF037966 | KY963753 |
|  | 18 | *Ardisia chinensis* 3 | MSZ-M96 | MF045731 | MF045613 | MF037982 | KY963769 |
|  | 19 | *Ardisia chinensis* 4 | MSZ-XL90 | MF045737 | MF045622 | MF037988 | KY963778 |
|  | 20 | *Ardisia chinensis* 5 | MSZ-XX89 | MF045738 | MF045623 | MF037989 | KY963781 |
|  | 21 | *Ardisia chinensis* 6 | MSZ-XZ25 | MF045741 | MF045626 | MF037992 | KY963784 |
| Ⅵ | 22 | *Ardisia corymbifera* 1 | MSZ-M150 | MF045681 | MF045563 | MF037932 | KY963719 |
|  | 23 | *Ardisia corymbifera* 2 | MSZ-M162 | MF045686 | MF045568 | MF037937 | KY963724 |
| Ⅶ | 24 | *Ardisia corymbifera* var. *tuberifera* 1 | MSZ-M200 | MF045703 | MF045585 | MF037954 | KY963741 |
|  | 25 | *Ardisia corymbifera* var. *tuberifera* 2 | MSZ-M212 | MF045706 | MF045588 | MF037957 | KY963744 |
|  | 26 | *Ardisia corymbifera* var. *tuberifera* 3 | MSZ-M194 | MF045700 | MF045582 | MF037951 | KY963738 |
|  | 27 | *Ardisia corymbifera* var. *tuberifera* 4 | MSZ-KG48 | MF045650 | MF045532 | MF037901 | KY963688 |
|  | 28 | *Ardisia corymbifera* var. *tuberifera* 5 | MSZ-M191 | MF045698 | MF045580 | MF037949 | KY963736 |
|  | 29 | *Ardisia corymbifera* var. *tuberifera* 6 | MSZ-M330 | MF045714 | MF045596 | MF037965 | KY963752 |
| Ⅷ | 30 | *Ardisia crenata* 1 | MSZ-M135 | MF045675 | MF045557 | MF037926 | KY963713 |
|  | 31 | *Ardisia crenata* 2 | MSZ-QZS2 | MF045733 | MF045615 | MF037984 | KY963771 |
|  | 32 | *Ardisia crenata* 3 | MSZ-M129 | MF045671 | MF045553 | MF037922 | KY963709 |
|  | 33 | *Ardisia crenata* 4 | MSZ-M103 | MF045658 | MF045540 | MF037909 | KY963696 |
|  | 34 | *Ardisia crenata* 5 | MSZ-M102 | MF045657 | MF045539 | MF037908 | KY963695 |
|  | 35 | *Ardisia crenata* 6 | MSZ-HZ318 | MF045644 | MF045526 | MF037895 | KY963682 |
|  | 36 | *Ardisia crenata* 7 | MSZ-M101 | MF045656 | MF045538 | MF037907 | KY963694 |
|  | 37 | *Ardisia crenata* 8 | MSZ-M345 | MF045723 | MF045605 | MF037974 | KY963761 |
| Ⅸ | 38 | *Ardisia crispa* 1 | MSZ-XBL29 | MF045635 | MF045621 | MF037987 | KY963777 |
|  | 39 | *Ardisia crispa* 2 | MSZ-BLJ46 | MF045736 | MF045517 | MF037886 | KY963673 |
| Ⅹ | 40 | *Ardisia cymosa* 1 | MSZ-M114 | MF045662 | MF045544 | MF037913 | KY963700 |
|  | 41 | *Ardisia cymosa* 2 | MSZ-M121 | MF045666 | MF045548 | MF037917 | KY963704 |
|  | 42 | *Ardisia cymosa* 3 | MSZ-M112 | MF045661 | MF045543 | MF037912 | KY963699 |
|  | 43 | *Ardisia cymosa* 4 | MSZ-5247 | MF045633 | MF045514 | MF037884 | KY963670 |
| XI | 44 | *Ardisia depressa* 1 | MSZ-M159 | MF045685 | MF045567 | MF037936 | KY963723 |
|  | 45 | *Ardisia depressa* 2 | MSZ-LS1 | MF045652 | MF045534 | MF037903 | KY963690 |
| XII | 46 | *Ardisia elegans* 1 | MSZ-LSM3 | MF045653 | MF045535 | MF037904 | KY963691 |
|  | 47 | *Ardisia elegans* 2 | MSZ-M335 | MF045717 | MF045599 | MF037968 | KY963755 |
|  | 48 | *Ardisia elegans* 3 | MSZ-M342 | MF045720 | MF045602 | MF037971 | KY963758 |
| XIII | 49 | *Ardisia ensifolia* 1 | MSZ-JYZJN | MF045649 | MF045531 | MF037900 | KY963687 |
|  | 50 | *Ardisia ensifolia* 2 | MSZ-M169 | MF045688 | MF045570 | MF037939 | KY963726 |
|  | 51 | *Ardisia ensifolia* 3 | MSZ-JY34 | MF045648 | MF045530 | MF037899 | KY963686 |
|  | 52 | *Ardisia ensifolia* 4 | MSZ-M182 | MF045693 | MF045575 | MF037944 | KY963731 |
| XIV | 53 | *Ardisia faberi* 1 | MSZ-M108 | MF045659 | MF045541 | MF037910 | KY963697 |
|  | 54 | *Ardisia faberi* 2 | MSZ-YYH26 | MF045743 | MF045628 | MF037994 | KY963786 |
| XV | 55 | *Ardisia filiformis* 1 | MSZ-M222 | MF045712 | MF045594 | MF037963 | KY963750 |
|  | 56 | *Ardisia filiformis* 2 | MSZ-M333 | MF045716 | MF045598 | MF037967 | KY963754 |
|  | 57 | *Ardisia filiformis* 3 | MSZ-M340 | MF045719 | MF045601 | MF037970 | KY963757 |
| XVI | 58 | *Ardisia fordii* 1 | MSZ-HS50 | MF045642 | MF045524 | MF037893 | KY963680 |
|  | 59 | *Ardisia fordii* 2 | MSZ-HS7 | MF045643 | MF045525 | MF037894 | KY963681 |
|  | 60 | *Ardisia fordii* 3 | MSZ-M193 | MF045699 | MF045581 | MF037950 | KY963737 |
|  | 61 | *Ardisia fordii* 4 | MSZ-M221 | MF045711 | MF045593 | MF037962 | KY963749 |
|  | 62 | *Ardisia fordii* 5 | MSZ-M73 | MF045727 | MF045609 | MF037978 | KY963765 |
| XVII | 63 | *Ardisia gigantifolia* 1 | MSZ-M343 | MF045721 | MF045603 | MF037972 | KY963759 |
|  | 64 | *Ardisia gigantifolia* 2 | MSZ-M80 | MF045730 | MF045612 | MF037981 | KY963768 |
|  | 65 | *Ardisia gigantifolia* 3 | MSZ-M128 | MF045670 | MF045552 | MF037921 | KY963708 |
|  | 66 | *Ardisia gigantifolia* 4 | MSZ-M127 | MF045669 | MF045551 | MF037920 | KY963707 |
|  | 67 | *Ardisia gigantifolia* 5 | MSZ-M118 | MF045665 | MF045547 | MF037916 | KY963703 |
|  | 68 | *Ardisia gigantifolia* 6 | MSZ-M217 | MF045708 | MF045590 | MF037959 | KY963746 |
|  | 69 | *Ardisia gigantifolia* 7 | MSZ-ZMT53 | MF045746 | MF045632 | MF037997 | KY963790 |
| XVIII | 70 | *Ardisia hancana* 1 | MSZ-M177 | MF045691 | MF045573 | MF037942 | KY963729 |
|  | 71 | *Ardisia hancana* 2 | MSZ-M189 | MF045697 | MF045579 | MF037948 | KY963735 |
|  | 72 | *Ardisia hancana* 3 | MSZ-M145 | MF045679 | MF045561 | MF037930 | KY963717 |
|  | 73 | *Ardisia hancana* 4 | MSZ-DLS10 | MF045639 | MF045521 | MF037890 | KY963677 |
| XIX | 74 | *Ardisia humilis* 1 | MSZ-AZ16 |  | MF045516 |  | KY963672 |
|  | 75 | *Ardisia humilis* 2 | MSZ-ZJN8 |  | MF045630 |  | KY963788 |
| XX | 76 | *Ardisia japonica* 1 | MSZ-M138 | MF045677 | MF045559 | MF037928 | KY963715 |
|  | 77 | *Ardisia japonica* 2 | MSZ-ZJN21 | MF045744 | MF045629 | MF037995 | KY963787 |
|  | 78 | *Ardisia japonica* 3 | MSZ-M137 | MF045676 | MF045558 | MF037927 | KY963714 |
| XXI | 79 | *Ardisia linangensis* 1 | MSZ-LN17 | MF045651 | MF045533 | MF037902 | KY963689 |
|  | 80 | *Ardisia linangensis* 2 | MSZ-M147 | MF045680 | MF045562 | MF037931 | KY963718 |
| XXII | 81 | *Ardisia maclurei* 1 | MSZ-M126 | MF045668 | MF045550 | MF037919 | KY963706 |
|  | 82 | *Ardisia maclurei* 2 | MSZ-XY38 | MF045740 | MF045625 | MF037991 | KY963783 |
| XXIII | 83 | *Ardisia mamillata* 1 | MSZ-M124 | MF045667 | MF045549 | MF037918 | KY963705 |
|  | 84 | *Ardisia mamillata* 2 | MSZ-M97 | MF045732 | MF045614 | MF037983 | KY963770 |
|  | 85 | *Ardisia mamillata* 3 | MSZ-BMZ36 | MF045637 | MF045519 | MF037888 | KY963675 |
| XXIV | 86 | *Ardisia neriifolia* 1 | MSZ-M170 | MF045689 | MF045571 | MF037940 | KY963727 |
|  | 87 | *Ardisia neriifolia* 2 | MSZ-M184 | MF045694 | MF045576 | MF037945 | KY963732 |
|  | 88 | *Ardisia neriifolia* 3 | MSZ-D14 | MF045638 | MF045520 | MF037889 | KY963676 |
| XXV | 89 | *Ardisia omissa* 1 | MSZ-GE30 | MF045640 | MF045522 | MF037891 | KY963678 |
|  | 90 | *Ardisia omissa* 2 | MSZ-HLZ19 | MF045641 | MF045523 | MF037892 | KY963679 |
|  | 91 | *Ardisia omissa* 3 | MSZ-M142 | MF045678 | MF045560 | MF037929 | KY963716 |
|  | 92 | *Ardisia omissa* 4 | MSZ-M188 | MF045696 | MF045578 | MF037947 | KY963734 |
|  | 93 | *Ardisia omissa* 5 | MSZ-M195 | MF045701 | MF045583 | MF037952 | KY963739 |
| XXVI | 94 | *Ardisia primulifolia* 1 | MSZ-M134 | MF045674 | MF045556 | MF037925 | KY963712 |
|  | 95 | *Ardisia primulifolia* 2 | MSZ-M348 | MF045725 | MF045607 | MF037976 | KY963763 |
|  | 96 | *Ardisia primulifolia* 3 | MSZ-BLZ9 | MF045636 | MF045518 | MF037887 | KY963674 |
| XXVII | 97 | *Ardisia pusilla* 1 | MSZ-M100 | MF045655 | MF045537 | MF037906 | KY963693 |
|  | 98 | *Ardisia pusilla* 2 | MSZ-M77 | MF045728 | MF045610 | MF037979 | KY963766 |
|  | 99 | *Ardisia pusilla* 3 | MSZ-JJH35 | MF045647 | MF045529 | MF037898 | KY963685 |
| XXVIII | 100 | *Ardisia quinquegona* 1 | MSZ-M216 | MF045707 | MF045589 | MF037958 | KY963745 |
|  | 101 | *Ardisia quinquegona* 2 | MSZ-M220 | MF045710 | MF045592 | MF037961 | KY963748 |
|  | 102 | *Ardisia quinquegona* 3 | MSZ-M164 | MF045687 | MF045569 | MF037938 | KY963725 |
|  | 103 | *Ardisia quinquegona* 4 | MSZ-LSS55 | MF045654 | MF045536 | MF037905 | KY963692 |
|  | 104 | *Ardisia quinquegona* 5 | MSZ-M337 | MF045718 | MF045600 | MF037969 | KY963756 |
| XXIX | 105 | *Ardisia replicata* 1 | MSZ-JBZ57 | MF045645 | MF045527 | MF037896 | KY963683 |
|  | 106 | *Ardisia replicata* 2 | MSZ-M173 | MF045690 | MF045572 | MF037941 | KY963728 |
|  | 107 | *Ardisia replicata* 3 | MSZ-M179 | MF045692 | MF045574 | MF037943 | KY963730 |
| XXX | 108 | *Ardisia solanacea* 1 | MSZ-STC |  | MF045617 |  | KY963773 |
|  | 109 | *Ardisia solanacea* 2 | MSZ-STC1 |  | MF045618 |  | KY963774 |
|  | 110 | *Ardisia solanacea* 3 | MSZ-STC5 |  | MF045619 |  | KY963775 |
| XXXI | 111 | *Ardisia velutina* 1 | MSZ-M154 | MF045683 | MF045565 | MF037934 | KY963721 |
|  | 112 | *Ardisia velutina* 2 | MSZ-M158 | MF045684 | MF045566 | MF037935 | KY963722 |
|  | 113 | *Ardisia velutina* 3 | MSZ-ZM5 | MF045745 | MF045631 | MF037996 | KY963789 |
| XXXII | 114 | *Ardisia villosa* 1 | MSZ-M218 | MF045709 | MF045591 | MF037960 | KY963747 |
|  | 115 | *Ardisia villosa* 2 | MSZ-M227 | MF045713 | MF045595 | MF037964 | KY963751 |
|  | 116 | *Ardisia villosa* 3 | MSZ-M344 | MF045722 | MF045604 | MF037973 | KY963760 |
|  | 117 | *Ardisia villosa* 4 | MSZ-M349 | MF045726 | MF045608 | MF037977 | KY963764 |
|  | 118 | *Ardisia villosa* 5 | MSZ-XXH27 | MF045739 | MF045624 | MF037990 | KY963782 |
|  | 119 | *Ardisia villosa* 6 | MSZ-YXH28 | MF045742 | MF045627 | MF037993 | KY963785 |
| XXXII | 120 | *Ardisia virens* 1 | MSZ-M153 | MF045682 | MF045564 | MF037933 | KY963720 |
|  | 121 | *Ardisia virens* 2 | MSZ-M185 | MF045695 | MF045577 | MF037946 | KY963733 |
|  | 122 | *Embelia laeta* | MSZ-STZ | MN381723 | MN381719 | MN381721 | MN381717 |
|  | 123 | *Embelia rudis* | MSZ-WMSTZ | MN381724 | MN381720 | MN381722 | MN381718 |
|  | 123 | *Glaux maritima* | * | AF213821 | JN895983 | JN044745 | JF976473 |

Asterisks (*) indicate that the sequences were obtained from GenBank.
