# Supplementary material for: Evaluation of Four Commonly Used DNA Barcoding Loci for Ardisia Species Identification
Source: Front Plant Sci. 2022 Apr 7;13:860778. doi: 10.3389/fpls.2022.860778 (PMC9021757; doi:10.3389/fpls.2022.860778)
Supplement: Supplementary file 1 [file Data_Sheet_1.ZIP › Revised Supplementary files/Supplementary Table 2.doc]

**Supplementary Table 2** Sequences of the primers used in this study.

| Sequence Name | Primers | Sequence (5’ to 3’) |
| --- | --- | --- |
| ITS | P1 | CGTAACAAGGTTTCCGTAGGTGAA |
|  | P2 | TTATTGATATGCTTAAACTCAGCGGG |
| *psb*A-*trn*H | PA | GTTATGCATGAACGTAATGCTC |
|  | TH | CGCGCATGGTGGATTCACAATCC |
| *mat*K | 390F | CGATCTATTCATTCAATATTTC |
|  | 1326R | TCTAGCACACGAAAGTCGAAGT |
| *rbc*L | 1f | ATGTCACCACAAACAGAAAC |
|  | 724r | TCGCATGTACCTGCAGTAGC |
