# Supplementary material for: Evaluation of Four Commonly Used DNA Barcoding Loci for Ardisia Species Identification
Source: Front Plant Sci. 2022 Apr 7;13:860778. doi: 10.3389/fpls.2022.860778 (PMC9021757; doi:10.3389/fpls.2022.860778)
Supplement: Supplementary file 1 [file Data_Sheet_1.ZIP › Revised Supplementary files/Supplementary Table 3.doc]

**Supplementary Table 3 Identification success rates based on analysis of the ‘Best March’ and ‘Best Close Match’ functions of TAXONDNA software for each single candidate barcodes and combinations of them.**

|  |  | Best match | | |  | Best close match | | | | Threshold,% |
| --- | --- | --- | --- | --- | --- | --- | --- | --- | --- | --- |
|  | No. | Correct | Ambiguous | Incorrect |  | Correct | Ambiguous | Incorrect | No match |  |
| ITS | 121 | 115 (95.04%) | 3 (2.47%) | 3 (2.47%) |  | 115 (95.04%) | 2 (1.65%) | 1 (0.82%) | 25 (20.66%) | 0.15 |
| *mat*K | 119 | 52 (43.69%) | 66 (55.46%) | 1 (0.84%) |  | 52 (43.69%) | 65 (54.62%) | 1 (0.84%) | 1 (0.84%) | 0.23 |
| *psb*A-*tm*H | 114 | 78 (68.42%) | 34 (29.82%) | 2 (1.75%) |  | 78 (68.42%) | 34 (29.82%) | 2 (1.75%) | 0 (0.00%) | 3.55 |
| *rbc*L | 114 | 17 (14.91%) | 94 (82.45%) | 3 (2.63%) |  | 17 (14.91%) | 93 (81.57%) | 3 (2.63%) | 1 (0.87%) | 0.56 |
| ITS + *mat*K | 119 | 115 (96.63%) | 3 (2.52%) | 1 (0.84%) |  | 80 (67.22%) | 2 (1.68%) | 1 (0.84%) | 36 (30.25%) | 0.05 |
| ITS + *rbc*L | 114 | 107 (93.85%) | 3 (2.63%) | 4 (3.5%) |  | 76 (66.66%) | 2 (1.75%) | 1 (0.87%) | 35 (30.7%) | 0.07 |
| ITS + *psb*A-*tm*H | 114 | 106 (92.98%) | 5 (4.38%) | 3 (2.63%) |  | 79 (69.29%) | 0 (0.00%) | 2 (1.75%) | 33 (28.94%) | 0.07 |
| *mat*K + *psb*A-*tm*H | 114 | 87 (76.31%) | 25 (21.92%) | 2 (1.75%) |  | 87 (76.31%) | 25 (21.92%) | 2 (1.75%) | 0 (0.00%) | 1.36 |
| *mat*K + *rbc*L | 114 | 62 (54.38%) | 50 (43.85%) | 2 (1.75%) |  | 62 (54.38%) | 49 (42.98%) | 2 (1.75%) | 1 (0.87%) | 0.25 |
| *psb*A-*tm*H + *rbc*L | 114 | 89 (78.06%) | 19 (16.66%) | 6 (5.26%) |  | 89 (78.06%) | 19 (16.66%) | 6 (5.26%) | 1 (0.87%) | 1.69 |
| ITS + *mat*K + *psb*A-*tm*H | 114 | 107 (93.85%) | 4 (3.50%) | 3 (2.63%) |  | 70 (61.4%) | 0 (0.00%) | 2 (1.75%) | 42 (36.84%) | 0.03 |
| ITS + *mat*K +*rbc*L | 114 | 109 (95.61%) | 3 (2.63%) | 2 (1.75%) |  | 65 (57.01%) | 2 (1.75%) | 1 (0.87%) | 46 (40.35%) | 0.03 |
| ITS + *psb*A-*tm*H + *rbc*L | 114 | 104 (91.22%) | 7 (6.14%) | 3 (2.63%) |  | 68 (59.64%) | 0 (0.0%) | 2 (1.75%) | 44 (38.59%) | 0.05 |
| *mat*K + *psb*A-*tm*H + *rbc*L | 114 | 90 (78.94%) | 18 (15.78%) | 6 (5.26%) |  | 90 (78.94%) | 18 (15.78%) | 6 (5.26%) | 0 (0.00%) | 1.00 |
| ITS + *mat*K + *psb*A-*tm*H + *rbc*L | 114 | 105 (92.1%) | 6 (5.26%) | 3 (2.63%) |  | 59 (51.75%) | 0 (0.00%) | 2 (1.75%) | 53 (46.49%) | 0.02 |
