# Supplementary material for: Evaluation of Four Commonly Used DNA Barcoding Loci for Ardisia Species Identification
Source: Front Plant Sci. 2022 Apr 7;13:860778. doi: 10.3389/fpls.2022.860778 (PMC9021757; doi:10.3389/fpls.2022.860778)
Supplement: Supplementary file 1 [file Data_Sheet_1.ZIP › Revised Supplementary files/supplementary figure 2.doc]

Appendix S3 **The NJ tree based on *mat*K + *psb*A-*tm*H sequences. Successfully identified species are with bootstrap values above 60%. The dotted line indicates unsuccessful identified species.**
